# Circulating concentrations of biomarkers and metabolites related to vitamin status, one-carbon and the kynurenine pathways in US, Nordic, Asian, and Australian populations^1^,^2^,^3^

**DOI:** 10.3945/ajcn.116.151241

**Published:** 2017-04-19

**Authors:** Øivind Midttun, Despoina Theofylaktopoulou, Adrian McCann, Anouar Fanidi, David C Muller, Klaus Meyer, Arve Ulvik, Wei Zheng, Xiao-Ou Shu, Yong-Bing Xiang, Ross Prentice, Cynthia A Thomson, Mary Pettinger, Graham G Giles, Allison Hodge, Qiuyin Cai, William J Blot, Jie Wu, Mikael Johansson, Johan Hultdin, Kjell Grankvist, Victoria L Stevens, Marjorie L McCullough, Stephanie J Weinstein, Demetrius Albanes, Arnulf Langhammer, Kristian Hveem, Marit Næss, Howard D Sesso, J Michael Gaziano, Julie E Buring, I-Min Lee, Gianluca Severi, Xuehong Zhang, Jiali Han, Meir J Stampfer, Stephanie A Smith-Warner, Anne Zeleniuch-Jacquotte, Loic le Marchand, Jian-Min Yuan, Lesley M Butler, Woon-Puay Koh, Renwei Wang, Yu-Tang Gao, Ulrika Ericson, Emily Sonestedt, Regina G Ziegler, Neal D Freedman, Kala Visvanathan, Miranda R Jones, Caroline Relton, Paul Brennan, Mattias Johansson, Per M Ueland

**Affiliations:** 4 Bevital AS, Bergen, Norway;; 5 Department of Clinical Science, University of Bergen, Bergen, Norway;; 6 Genetic Epidemiology Group, International Agency for Research on Cancer, Lyon, France;; 7 Department of Epidemiology and Biostatistics, Imperial College London, London, United Kingdom;; 8 Division of Epidemiology, Department of Medicine, Vanderbilt Epidemiology Center, Vanderbilt-Ingram Cancer Center, Vanderbilt University School of Medicine, Nashville, TN;; 9 Department of Epidemiology, Shanghai Cancer Institute, Renji Hospital, Shanghai Jiaotong University School of Medicine, Shanghai, China;; 10 Division of Public Health Sciences, Fred Hutchinson Cancer Research Center, Seattle, WA;; 11 Health Promotion Sciences, Mel and Enid Zuckerman College of Public Health, University of Arizona, Tucson, AZ;; 12 Cancer Epidemiology Center, Cancer Council Victoria, Melbourne, Victoria, Australia;; 13 Center for Epidemiology and Biostatistics, Melbourne School of Population and Global Health, University of Melbourne, Victoria, Australia;; 14 International Epidemiology Institute, Rockville, MD;; 15 Department of Radiation Sciences, Oncology, and; 16 Department of Medical Biosciences, Clinical Chemistry, Umeå University, Umeå, Sweden;; 17 Epidemiology Research Program, American Cancer Society, Atlanta, GA;; 18 Division of Cancer Epidemiology and Genetics, National Cancer Institute, NIH, Bethesda, MD;; 19 Nord-Trøndelag Health Study Research Center, Department of Public Health and Nursing, Faculty of Medicine and Health Science, Norwegian University of Science and Technology, Levanger, Norway;; 20 Divisions of Preventive Medicine and; 21 Aging, Brigham and Women’s Hospital, Boston, MA;; 22 Departments of Epidemiology and; 23 Nutrition, Harvard T.H. Chan School of Public Health, Boston, MA;; 24 VA Boston Healthcare System, Boston, MA;; 25 Human Genetics Foundation, Turin, Italy;; 26 Centre for Research in Epidemiology and Population Health (U1018 French National Institute of Health and Medical Research), Facultés de Médecine Université Paris-Sud, Université de Versailles Saint-Quentin-en-Yvelines, Université Paris-Saclay, Villejuif, France;; 27 Channing Division of Network Medicine, Department of Medicine, Brigham and Women’s Hospital and Harvard Medical School, Boston, MA;; 28 Department of Population Health, New York University School of Medicine, New York, NY;; 29 Epidemiology Program, University of Hawaii Cancer Center, Honolulu, HI;; 30 Division of Cancer Control and Population Sciences, University of Pittsburgh Cancer Institute, Pittsburgh, PA;; 31 Department of Epidemiology, Graduate School of Public Health, University of Pittsburgh, Pittsburgh, PA;; 32 Duke–National University of Singapore (NSU) Medical School, Singapore, and Saw Swee Hock School of Public Health, NSU, Singapore, Singapore;; 33 Department of Epidemiology, Shanghai Cancer Institute, Shanghai Jiaotong University, Shanghai, China;; 34 Department of clinical sciences Malmö, Lund University, Lund, Sweden;; 35 Johns Hopkins Bloomberg School of Public Health and Johns Hopkins Sidney Kimmel Comprehensive Center, School of Medicine, Baltimore, MD;; 36 Institute of Genetic Medicine, Newcastle University, Newcastle, United Kingdom;; 37 Medical Research Council Integrative Epidemiology Unit, School of Social and Community Medicine, University of Bristol, Bristol, United Kingdom; and; 38 Laboratory of Clinical Biochemistry, Haukeland University Hospital, Bergen, Norway

**Keywords:** biomarker, Lung Cancer Cohort Consortium, one-carbon metabolism, tryptophan metabolism, vitamin status

## Abstract

**Background:** Circulating concentrations of biomarkers that are related to vitamin status vary by factors such as diet, fortification, and supplement use. Published biomarker concentrations have also been influenced by the variation across laboratories, which complicates a comparison of results from different studies.

**Objective:** We robustly and comprehensively assessed differences in biomarkers that are related to vitamin status across geographic regions.

**Design:** The trial was a cross-sectional study in which we investigated 38 biomarkers that are related to vitamin status and one-carbon and tryptophan metabolism in serum and plasma from 5314 healthy control subjects representing 20 cohorts recruited from the United States, Nordic countries, Asia, and Australia, participating in the Lung Cancer Cohort Consortium. All samples were analyzed in a centralized laboratory.

**Results:** Circulating concentrations of riboflavin, pyridoxal 5′-phosphate, folate, vitamin B-12, all-*trans* retinol, 25-hydroxyvitamin D, and α-tocopherol as well as combined vitamin scores that were based on these nutrients showed that the general B-vitamin concentration was highest in the United States and that the B vitamins and lipid soluble vitamins were low in Asians. Conversely, circulating concentrations of metabolites that are inversely related to B vitamins involved in the one-carbon and kynurenine pathways were high in Asians. The high B-vitamin concentration in the United States appears to be driven mainly by multivitamin-supplement users.

**Conclusions:** The observed differences likely reflect the variation in intake of vitamins and, in particular, the widespread multivitamin-supplement use in the United States. The results provide valuable information about the differences in biomarker concentrations in populations across continents.

## INTRODUCTION

The quantitative measurement of circulating biomarker concentrations has been used in various studies that have investigated nutritional status, vitamin status, and lifestyle factors in relation to mortality and morbidities such as cancer and cardiovascular disease.

Circulating concentrations of vitamins and associated metabolites are related to vitamin intakes (1–3), which vary across the globe because of factors such as diet, lifestyle, vitamin-enrichment and food-fortification practices, and supplement use. In some countries, food fortification with various vitamins has been implemented to correct identified deficiencies or to reduce disease risk. In the United States, fortification has become widespread; margarine and milk have been voluntarily fortified with vitamins A and D since the 1930s (4, 5), while enrichment of flour and cereals with thiamin, riboflavin, and niacin since the 1940s (5) and with folic acid since 1998 (5) has been mandatory. In comparison, other countries generally have a much more restrictive approach to vitamin fortification and have implemented voluntary rather than mandatory food-fortification strategies. In addition to the mandatory fortification strategies in the United States, food manufacturers often add various vitamins to different food products (6–8), occasionally at very high concentrations (9), on a discretionary basis and at times as a marketing approach to promote product sales. The individual use of vitamin supplements adds to these geographical differences in vitamin intake from enriched and fortified foods.

Metabolism of the amino acids methionine (10) and tryptophan (11) are dependent on various B vitamins serving as cofactors. Thus, intakes (12, 13) and circulating concentrations (14, 15) of B vitamins can also influence the concentrations of these amino acids and their downstream metabolites.

The performance of different analytical methods used to quantify biomarkers also varies (16, 17), which has further contributed to the inherent challenge of comparing results between studies. The use of a centralized laboratory can overcome such difficulties.

Based on the European analyses that showed inverse relations between circulating vitamin B-6 [pyridoxal 5′-phosphate (PLP)]^39^ and methionine and lung cancer risk (18), the Lung Cancer Cohort Consortium (LC3) was established to prospectively investigate associations between vitamin B-6, one-carbon metabolites, and related biomarkers and lung cancer in a large number of cohorts across different geographic regions. In the current investigation, we describe circulating concentrations of 38 biomarkers that are related to vitamin status, one-carbon metabolism (OCM), and tryptophan metabolism (through the kynurenine pathway) in the 5314 healthy control subjects from the 20 participating cohorts of the LC3, which has a total of 10,728 participants from the United States, Nordic region, Asia, and Australia. The inclusion of 7 circulating vitamins allowed for the construction of composite vitamin scores to describe general vitamin status across geographic regions. All samples underwent identical biochemical analyses with the use of the same analytic assays in a single laboratory.

## METHODS

### Study design and population

**FIGURE 1:**
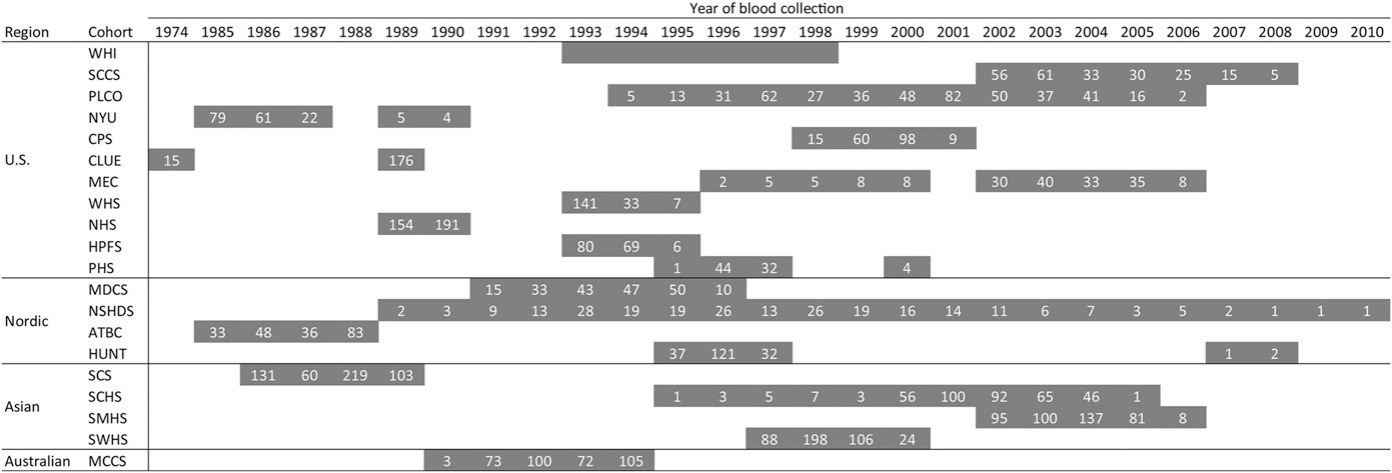
Year of blood sample collection. Numbers in each cell indicate the number of samples included from each cohort that year; for the WHI cohort, this information was not available. ATBC, Alpha-Tocopherol, Beta-Carotene Cancer Prevention Study; CLUE, Campaign Against Cancer and Stroke and Campaign Against Cancer and Heart Disease; CPS, American Cancer Society Cancer Prevention Study-II Nutrition Cohort; HPFS, Health Professionals Follow-Up Study; HUNT, Nord-Trøndelag Health Study; MCCS, Melbourne Collaborative Cohort Study; MDCS, Malmö Diet and Cancer Study; MEC, Multiethnic Cohort; NHS, Nurses’ Health Study; NSHDS, Northern Sweden Health and Disease Study Cohort; NYU, New York University Women’s Health Study; PHS, Physicians’ Health Study; PLCO, Prostate, Lung, Colorectal and Ovarian Cancer Screening Trial; SCCS, Southern Community Cohort Study; SCHS, Singapore Chinese Health Study; SCS, Shanghai Cohort Study; SMHS, Shanghai Men’s Health Study; SWHS, Shanghai Women’s Health Study; U.S., United States; WHI, Women’s Health Initiative; WHS, Women’s Health Study.

Information on the participating cohorts, including cohort acronyms, is shown in **Supplemental Methods**. Study participants included the 5364 healthy control subjects from the LC3. The consortium consisted of 20 cohorts: 11 cohorts from the United States, 4 cohorts from the Nordic region (Norway, Sweden, and Finland), 4 cohorts from Asia (Chinese populations residing in Shanghai or Singapore), and 1 cohort from Australia. Each cohort contributed 81–513 control participants. Blood samples (serum or plasma) were collected from 1974 to 2010 (**Figure 1**). We excluded 50 participants with missing biomarker concentrations in plasma or serum samples, which provided a study population of 5314 participants with a complete data set (**Supplemental Figure 1**). Demographic data for the total study population and each geographic region are shown in **Table 1** and, for each cohort, in **Supplemental Table 1**. All participants gave written informed consent to participate in the study. The research was approved by the institutional review board of the International Agency for Research of Cancer and each participating cohort.

**TABLE 1:** Baseline characteristics of study population by geographic region^1^

		Region					United States by MV use		
	Total	United States	Nordic	Asian	Australian	*P*	No	Yes	*P*
*n*	5314	2397	835	1729	353		1440	840	
Sex, F, *n* (%)	2422 (45.6)	1406 (58.7)	360 (43.1)	515 (29.8)	141 (39.9)	<0.001	797 (55.3)	555 (66.1)	<0.001
Age,^2^ y	62.0 (47.0–75.0)	68.0 (53.0–79.0)	59.9 (44.6–70.8)	63.0 (46.5–75.0)	61.0 (45.0–67.4)	<0.001	64.0 (47.8–77.0)	64.1 (47.0–79.0)	0.012
Education, *n* (%)						<0.001			<0.001
Less than high school	1655 (31.4)	215 (9.1)	365 (44.2)	843 (49.0)	232 (65.7)		160 (11.6)	53 (6.5)	
Completed high school	781 (14.8)	374 (15.8)	143 (17.3)	227 (13.2)	37 (10.5)		234 (16.9)	127 (15.5)	
Vocational school	912 (17.3)	435 (18.3)	167 (20.2)	277 (16.1)	33 (9.3)		273 (19.7)	140 (17.1)	
Some college	715 (13.6)	390 (16.4)	120 (14.5)	196 (11.4)	9 (2.5)		234 (16.9)	141 (17.2)	
College graduate	496 (9.4)	319 (13.5)	22 (2.7)	113 (6.6)	42 (11.9)		195 (14.1)	106 (13.0)	
Graduate studies	636 (12.1)	564 (23.8)	8 (1.0)	64 (3.7)	0 (0.0)		289 (20.9)	251 (30.7)	
Unknown	73 (1.4)	73 (3.1)	0 (0.0)	0 (0.0)	0 (0.0)		46 (3.3)	11 (1.3)	
Smoking status, *n* (%)						<0.001			0.015
Never	1286 (24.2)	569 (23.7)	107 (12.8)	561 (32.4)	49 (13.9)		323 (22.4)	228 (27.1)	
Former	1517 (28.5)	1006 (42.0)	190 (22.8)	176 (10.2)	145 (41.1)		607 (42.1)	343 (40.8)	
Current	2511 (47.3)	822 (34.3)	538 (64.4)	992 (57.4)	159 (45.0)		511 (35.5)	269 (32.0)	
BMI,^3^ kg/m^2^	24.7 (19.3–33.4)	25.8 (20.6–35.6)	25.8 (20.2–33.2)	24.1 (18.9–32.6)	27.5 (21.2–35.6)	<0.001	25.8 (20.1–35.6)	25.1 (20.1–34.2)	0.011
MV use, all participants, *n* (%)						<0.001	—	—	—
Never	527 (9.9)	527 (22.0)	0 (0.0)	0 (0.0)	0 (0.0)		—	—	
Ever	329 (6.2)	329 (13.7)	0 (0.0)	0 (0.0)	0 (0.0)		—	—	
No current	2149 (40.4)	914 (38.1)	150 (18.0)	779 (45.1)	306 (86.7)		—	—	
Current	671 (12.6)	511 (21.3)	55 (6.6)	58 (3.4)	47 (13.3)		—	—	
Missing	1638 (30.8)	116 (4.8)	630 (75.4)	892 (51.6)	0 (0.0)		—	—	
MV use in subjects with available data, *n* (%)						<0.001	—	—	—
No (never + no current)	2676 (72.8)	1441 (63.2)	150 (73.2)	779 (93.1)	306 (86.7)		—	—	
Yes (ever + current)	1000 (27.2)	840 (36.8)	55 (26.8)	58 (6.9)	47 (13.3)		—	—	

1 *P* values were determined with the use of a Kruskal-Wallis, ANOVA, or chi-square test. MV, multivitamin supplement.

2 All values are means (5th–95th percentiles).

3 All values are medians (5th–95th percentiles). BMI was calculated as weight divided by the square of height.

### Multivitamin-supplement use and smoking

Data regarding the self-reported use of multivitamin-supplements (defined as supplements that contained ≥3 vitamins) were obtained from questionnaires and were coded as current or no-current use for 12 cohorts and as ever or never for 4 (United States) cohorts (Supplemental Table 1). In the United States, circulating vitamin concentrations were similar between subjects who reported current use of multivitamin supplements and those who reported ever use of multivitamin supplements (data not shown). Similar vitamin concentrations were also found for those reporting no-current use compared with those reporting never use (data not shown). Therefore, we combined subjects who reported current and ever use into multivitamin-supplement users and those who reported no-current or never use into nonusers. No information on multivitamin-supplement use was available for this study from 2 Nordic and 2 Asian cohorts and for a varying number of participants in several of the other cohorts (Supplemental Table 1). Smoking was classified via self-reports as never, former, or current smoker.

### Biochemical analyses

All plasma and serum samples were stored at ≤−80°C from the time of collection until shipment to the Bevital laboratory (www.bevital.no) for biochemical analyses. Plasma concentrations of methionine, total homocysteine (tHcy), cystathionine, total cysteine, serine, glycine, sarcosine, methylmalonic acid (MMA), tryptophan, and kynurenine were measured with the use of gas chromatography–tandem mass spectrometry (19). Methionine sulfoxide (MetSO), choline, betaine, dimethylglycine, creatinine, arginine, asymmetric dimethylarginine, symmetric dimethylarginine, homoarginine (20), PLP, pyridoxal, 4-pyridoxic acid, riboflavin, kynurenic acid, anthranilic acid, 3-hydroxykynurenine (HK), xanthurenic acid (XA), 3-hydroxyanthranilic acid, quinolinic acid, cotinine (21), all-*trans* retinol (vitamin A), 25-hydroxyvitamin D_2_ [25(OH)D_2_], 25(OH)D_3_, α-tocopherol, and γ-tocopherol (22) were analyzed with the use of liquid-chromatography–tandem mass spectrometry. Folate (23) and vitamin B-12 (24) were determined by microbiological methods, whereas C-reactive protein was analyzed with the use of an immunomatrix-assisted laser-desorption ionization–mass spectrometry (25). A plasma sample was included as a quality control in all batches.

We modeled the seasonality of circulating 25(OH)D_3_ separately for each cohort with the use of a function that included 2 pairs of sine and cosine functions of the day of blood collection. The sum of 25(OH)D_2_ and season-adjusted 25(OH)D_3_ was combined into season-adjusted total 25(OH)D, which was used as a measure of vitamin D status. Because methionine may be oxidized to MetSO during sample storage (26), we used total methionine (i.e., methionine plus MetSO) as a measure of circulating methionine concentrations. The kynurenine-to-tryptophan ratio was calculated as kynurenine (expressed in nmol/L) divided by tryptophan (expressed as μmol/L), and the PAr was calculated as 4-pyridoxic acid:(PLP plus pyridoxal) (27).

### Statistical methods

Because most biomarkers were not normally distributed, crude circulating biomarker concentrations are reported as geometric means (5th and 95th percentiles). Values of cotinine, which is a marker of recent nicotine exposure, less than the limit of detection (1 nmol/L) were set to 1 nmol/L (which is well below the concentrations in both passive and active smokers) before being log transformed. Geometric means (95% CIs) by region were estimated with the use of mixed models that were adjusted for age, sex, and smoking status (former compared with never; current compared with never) with the cohort as a random effect. Between-region spreads of adjusted biomarker geometric means were calculated as CVs (SD divided by the mean of the geometric means, expressed as %). In the US region, we also investigated biomarker concentrations after stratification by multivitamin-supplement use (by combining current and ever compared with no-current and never), and prefolate fortification compared with postfolate fortification (1998). The effect of multivitamin-supplement use was not investigated for non-US populations because such information was only available for a low number of these participants. Geometric means (95% CIs) by cohort were estimated by adjusting for age, sex, and smoking status (former compared with never; current compared with never) with the use of generalized linear models.

We investigated proportional differences at each fifth percentile of biomarker distributions across the regions (United States, Nordic region, Asia, and Australia) by quantile regression (28). The results were plotted graphically as the percentage of differences between regions (with the Unites States as the reference) compared with the metabolite concentrations at each quantile cutoff. These models were adjusted for age (years) at blood sampling, sex, smoking (former compared with never; current compared with never) and cohort.

Patterns in biomarker concentrations across cohorts were investigated by performing a principal component analysis (PCA) on a matrix that contained centered and standardized cohort geometric mean biomarker concentrations from the generalized linear models. To ensure vitamin B-6 was weighted in the same way as other biomarkers were weighted, we included only one of the analyzed vitamin B-6 forms [i.e., PLP, which is the most commonly used vitamin B-6 marker (29)] in the PCA.

ANOVA was used for comparisons of normally distributed variables, and the Kruskal-Wallis test was used for comparisons of variables that were not normally distributed. Categorical variables were compared by using the chi-square test.

We combined individual circulating vitamin concentrations, which were log transformed, centered and standardized to have a mean of 0 and SD of 1, into 3 different vitamin scores to obtain measures of general vitamin status. Thus, the B-vitamin score (BVS) was obtained as the mean of the transformed concentrations of riboflavin, PLP, folate, and vitamin B-12. The fat-soluble vitamin score (FVS) included vitamin A, 25(OH)D, and α-tocopherol. The total vitamin score (TVS) combined all 7 vitamins. Each combined vitamin score was again standardized to have a mean of 0 and SD of 1. Only the vitamin E form α-tocopherol was included in the FVS and TVS because this is the form that is usually used for the assessment of vitamin E status (30). Vitamin scores across regions, cohorts, and US multivitamin-supplement users and nonusers were estimated by adjusting for age, sex, and smoking as previously described for biomarker concentrations.

Statistical tests were 2-sided, and significance was determined at the 0.05 level. Statistical analyses were performed by using SPSS version 22 for Windows software (SPSS Inc.) and R version 3.2.3 software (http://www.r-project.org; The R Foundation) [using the lm function, the packages lme4 (31), lmerTest, quantreg, and prcomp].

## RESULTS

### Demographics

Demographic data are given in Table 1 and Supplemental Table 1. Overall, the proportion of women was 45.6%. The geometric mean age differed across regions [from 59.9 y (Nordic) to 68.0 y (United States); *P* < 0.001]. Of the total population, 47.3% were current smokers, ranging from 34.3% of subjects in the United States to 64.4% of subjects in the Nordic region.

For 30.8% of the total population, information on multivitamin-supplement use was missing mainly because such data were not available for this study from several Nordic and Asian cohorts (Table 1). Of subjects with this information available, 27.2% reported the use of such supplements, whereas 72.8% reported no use of such supplements. The prevalence of multivitamin-supplement use was 36.8% in the United States, 26.8% in the Nordic region (data available for only 205 participants), 6.9% in Asians, and 13.3% in Australians.

All samples from the United States were collected from 1985 to 2008 except for a small number of samples (*n* = 15) from the Campaign Against Cancer and Stroke and the Campaign Against Cancer and Heart Disease, which were collected in 1974. For the US-based Women’s Health Initiative cohort, information on the year of blood sampling for each participant was not available because of confidentiality concerns. Samples were collected from Nordic cohorts during 1985–2010, from Asian cohorts during 1986–2006, and from the Australian cohort during 1990–1994 (Figure 1). **Supplemental Table 2** shows the crude geometric mean (5th and 95th percentile) biomarker concentrations for the total population and each geographic region, whereas **Supplemental Tables 3** and **4** show these data for individual cohorts.

### PCA using cohort geometric means

**FIGURE 2:**
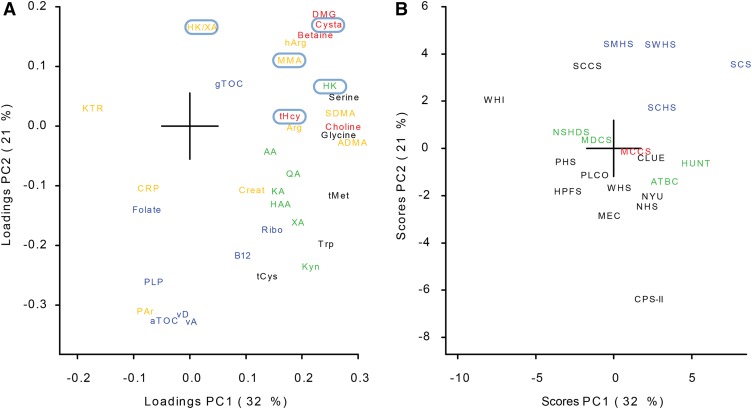
The first 2 PCs (PC1 and PC2) obtained from the principal component analysis based on centered and standardized geometric mean biomarker concentrations from all cohorts. The loading plot (A) shows the following colors and corresponding biomarkers: blue, vitamins; black, amino acids; red, one-carbon metabolites; green, kynurenines; and orange, other biomarkers. Functional B-vitamin markers are marked by light-blue ellipses. The score plot (B) shows the following colors and corresponding cohorts: black, United States; green, Nordic; blue, Asian; and red, Australian. AA, anthranilic acid; ADMA, asymmetric dimethylarginine; Arg, arginine; ATBC, Alpha-Tocopherol, Beta-Carotene Cancer Prevention Study; aTOC, α-tocopherol; B12, vitamin B-12; CLUE, Campaign Against Cancer and Stroke and Campaign Against Cancer and Heart Disease; CPS-II, American Cancer Society Cancer Prevention Study-II Nutrition Cohort; Creat, creatinine; CRP, C-reactive protein; Cysta, cystathionine; DMG, dimethylglycine; gTOC, γ-tocopherol; HAA, 3-hydroxyanthranilic acid; hArg, homoarginine; HK, 3-hydroxykynurenine; HPFS, Health Professionals Follow-Up Study; HUNT, Nord-Trøndelag Health Study; KA, kynurenic acid; KTR, kynurenine:tryptophan ratio; Kyn, kynurenine; MCCS, Melbourne Collaborative Cohort Study; MDCS, Malmö Diet and Cancer Study; MEC, Multiethnic Cohort; MMA, methylmalonic acid; NHS, Nurses’ Health Study; NSHDS, Northern Sweden Health and Disease Study Cohort; NYU, New York University Women’s Health Study; PAr, 4-pyridoxic acid:(pyridoxal 5′-phosphate plus pyridoxal); PC, principal component; PHS, Physicians’ Health Study; PLCO, Prostate, Lung, Colorectal and Ovarian Cancer Screening Trial; PLP, pyridoxal 5′-phosphate; QA, quinolinic acid; Ribo, riboflavin; SCCS, Southern Community Cohort Study; SCHS, Singapore Chinese Health Study; SCS, Shanghai Cohort Study; SDMA, symmetric dimethylarginine, SMHS, Shanghai Men’s Health Study; SWHS, Shanghai Women’s Health Study; tCys, total cysteine; tHcy, total homocysteine; tMet, total methionine; Trp, tryptophan; vA, vitamin A; vD, total 25-hydroxyvitamin D; WHI, Women’s Health Initiative; WHS, Women’s Health Study; XA, xanthurenic acid.

The first 2 PCs (**Figure 2**) explained a total of 53% (32% and 21%, respectively) of the variation in cohort geometric means obtained from mixed models. Vitamins (with the exception of γ-tocopherol) were grouped together with negative loadings on PC2 in the loading plot (Figure 2A). The functional B-vitamin markers (tHcy, MMA, cystathionine, HK, and HK:XA) and components of OCM were located opposite the vitamin group. The kynurenines (except HK) were also grouped together.

When the loading plots (Figure 2A) and score plots (Figure 2B) that were formed from the first 2 PCs were compared, the US cohorts were generally located in the same direction from the origin as the vitamin group and opposite the location of the functional B-vitamin markers. Asian cohorts were located in the same direction as the functional B-vitamin markers and OCM group and opposite the location of the US cohorts, whereas the Nordic and Australian cohorts were located closer to the center of the score plot.

PC3 and PC4 (which explained 12% and 8% of the variation in the data set, respectively) did not provide additional information about general vitamin concentrations or reveal other clear biomarker patterns and, thus, were not investigated further.

### Circulating vitamin scores

Vitamin scores across regions and cohorts are shown in **Table 2** and **Supplemental Tables 5** and **6**, respectively, and are shown graphically in **Supplemental Figure 2**. The highest mean BVS at 0.28 (95% CI: 0.10, 0.46; *P* < 0.05) was found in the United States, followed by the Australian (−0.09; 95% CI: −0.57, 0.39), Asian (−0.25; 95% CI: −0.53, 0.02), and Nordic (−0.28; 95% CI: −0.52, −0.03) regions. The FVS was highest at 0.48 (95% CI: 0.23, 0.72) in the Nordic region, followed by the Australian (0.40; 95% CI: −0.08, 0.86), US (0.06; 95% CI: −0.11, 0.25), and Asian (−0.32; 95% CI: −0.61, −0.07) regions. Across regions, the highest TVS was observed in the United States.

**TABLE 2:** Biomarker concentrations by region from mixed models^1^

	Region					United States by MV use	
	United States	Nordic	Asian	Australian	CV, %	No	Yes
*n*	2397	835	1729	353	—	1441	840
Vitamin score					—		
TVS	0.23 (0.07, 0.39)^2^	0.02 (−0.21, 0.24)^3^	−0.30 (−0.55, −0.05)^3^^,^^4^	0.08 (−0.35, 0.51)		−0.06 (−0.21, 0.08)	0.74 (0.59, 0.89)^5^
BVS	0.28 (0.10, 0.46)	−0.28 (−0.53, −0.03)^3^^,^^4^	−0.25 (−0.53, 0.02)^3^^,^^4^	−0.09 (−0.57, 0.39)^3^		−0.01 (−0.18, 0.17)	0.80 (0.62, 0.97)^5^
FVS	0.06 (−0.12, 0.23)	0.48 (0.24, 0.72)^5^	−0.32 (−0.59, −0.06)^3^^,^^6^	0.40 (−0.06, 0.87)		−0.13 (−0.29, 0.03)	0.41 (0.24, 0.57)^5^
Vitamin							
Vitamin B-2 (riboflavin), nmol/L	21.3 (17.5, 25.9)	17.0 (13.0, 22.3)	19.1 (14.1, 25.8)	25.2 (14.9, 42.6)	17	18.3 (15.0, 22.4)	28.0 (22.9, 34.3)^5^
PLP, nmol/L	50.1 (43.8, 57.3)	37.5 (31.1, 45.2)^3^	37.0 (30.1, 45.5)^3^	35.4 (24.8, 50.7)^3^	17	40.1 (35.7, 44.9)	72.3 (64.3, 81.3)^5^
Pyridoxal, nmol/L	17.0 (12.5, 23.1)	18.3 (11.9, 28.0)	15.9 (9.9, 25.6)	21.8 (9.5, 49.7)	14	13.1 (9.5, 17.9)	26.5 (19.3, 36.4)^5^
PA, nmol/L	31.8 (27.6, 36.6)	28.4 (23.4, 34.6)	18.0 (14.5, 22.4)^3^^–^^6^	24.0 (16.5, 34.9)	23	24.1 (21.1, 27.4)	50.3 (43.8, 57.6)^5^
Folate, nmol/L	28.5 (24.2, 33.6)	12.4 (9.8, 15.6)^3^^–^^5^	14.7 (11.4, 19.0)^3^^–^^5^	17.1 (11.0, 26.7)^3^	39	23.6 (19.9, 28.1)	38.1 (32.0, 45.4)^5^
Vitamin B-12, pmol/L	442 (409, 477)	457 (411, 508)	409 (363, 460)	407 (332, 500)	6	414 (385, 446)	491 (455, 529)^5^
Vitamin A (all-*trans* retinol), μmol/L	2.19 (2.10, 2.29)	2.37 (2.23, 2.52)	1.91 (1.79, 2.05)^3^^–^^6^	2.32 (2.06, 2.62)	9	2.15 (2.06, 2.24)	2.28 (2.18, 2.38)^5^
Vitamin D,^7^ nmol/L	53.0 (49.6, 56.6)	64.3 (58.6, 70.4)^4^^,^^5^	53.7 (48.5, 59.4)^6^	58.1 (48.8, 69.3)	9	49.0 (45.8. 52.5)	61.3 (57.2. 65.8)^5^
αTOC, μmol/L	31.4 (29.4, 33.6)	34.6 (31.5, 38.0)^5^	27.0 (24.3, 30.0)^3^^,^^6^^,^^8^	36.3 (30.3, 43.5)	13	29.3 (27.5, 31.1)	35.5 (33.3, 37.8)^5^
γTOC, μmol/L	4.38 (3.58, 5.35)	2.69 (2.03, 3.55)^5^	3.88 (2.84, 5.29)	1.69 (0.98, 2.91)^4^^,^^5^	38	5.00 (4.11, 6.08)	3.44 (2.83, 4.19)^5^
One-carbon metabolites							
tMet, μmol/L	27.4 (26.4, 28.5)	27.3 (25.9, 28.8)	29.7 (28.0, 31.5)	27.5 (24.8, 30.6)	4	27.6 (26.5, 28.8)	27.4 (26.3, 28.6)
tHcy, μmol/L	11.5 (10.6, 12.4)	11.0 (9.9, 12.3)	12.9 (11.4, 14.5)	12.8 (10.4, 15.7)	8	12.0 (11.1, 13.0)	10.8 (9.9, 11.7)^5^
Cystathionine, μmol/L	0.170 (0.157, 0.184)	0.190 (0.170, 0.212)^3^	0.283 (0.250, 0.319)^3^^–^^6^^,^^8^	0.185 (0.150, 0.229)	25	0.179 (0.165. 0.195)	0.157 (0.145. 0.171)^5^
tCys, μmol/L	300 (290, 311)	300 (286, 314)	286 (272, 302)	313 (286, 342)	4	300 (289, 310)	302 (292, 313)
Serine, μmol/L	107.6 (97.0, 119.4)	122.9 (106.3, 142.0)	136.4 (116.0, 160.4)	115.9 (87.5, 153.6)	10	110 (99, 122)	105 (95, 117)^5^
Glycine, μmol/L	265 (244, 287)	253 (226, 284)	278 (245, 315)	248 (199, 309)	5	266 (245, 289)	267 (245, 290)
Choline, μmol/L	12.8 (11.4, 14.4)	11.6 (9.8, 13.6)	14.3 (11.9, 17.2)	14.3 (10.4, 19.6)	10	12.9 (11.5, 14.6)	12.9 (11.4, 14.5)
Betaine, μmol/L	37.3 (34.7, 40.2)	32.3 (29.2, 35.8)	50.1 (44.8, 56.1)^3^^–^^6^^,^^8^	34.9 (28.6, 42.5)	20	37.7 (35.0, 40.6)	36.9 (34.2, 39.8)
Dimethylglycine, μmol/L	3.7 (3.4, 3.9)	4.0 (3.6, 4.4)	5.0 (4.5, 5.6)^3^^–^^6^^,^^8^	3.8 (3.2, 4.6)	15	3.75 (3.49, 4.04)	3.49 (3.24, 3.76)^5^
Tryptophan and metabolites							
Tryptophan, μmol/L	62.6 (59.3, 66.0)	67.6 (62.8, 72.8)	70.2 (64.7, 76.2)	67.5 (58.5, 77.9)	5	62.7 (59.4, 66.2)	62.8 (59.4, 66.3)
Kynurenine, μmol/L	1.47 (1.41, 1.53)	1.55 (1.47, 1.65)	1.58 (1.48, 1.68)	1.53 (1.37, 1.71)	3	1.48 (1.42, 1.55)	1.46 (1.40, 1.52)
KA, nmol/L	41.7 (39.1, 44.5)	40.7 (37.1, 44.5)	51.5 (46.6, 56.9)^^4^^,^^^6^	43.9 (36.9, 52.3)	11	41.8 (39.0, 44.7)	42.1 (39.2, 45.2)
AA, nmol/L	14.6 (13.5, 15.8)	14.9 (13.4, 16.6)	13.9 (12.3, 15.7)	15.6 (12.6, 19.2)	5	14.6 (13.5, 15.7)	14.7 (13.6, 15.9)
HK, nmol/L	33.9 (31.8, 36.2)	37.2 (34.0, 40.7)	40.6 (36.8, 44.9)^3^^,^^4^	37.6 (31.6, 44.8)	7	35.4 (33.2, 37.9)	31.8 (29.7, 34.0)^5^
XA, nmol/L	10.5 (9.6, 11.4)	12.4 (11.0, 13.9)	15.1 (13.2, 17.2)^3^^–^^5^	11.0 (8.7, 13.8)	17	10.53 (9.64, 11.49)	10.52 (9.60, 11.52)
HAA, nmol/L	27.1 (24.6, 29.9)	36.0 (31.5, 41.3)^3^^–^^5^	37.5 (32.3, 43.6)^3^^–^^5^	32.1 (24.7, 41.7)	14	26.6 (24.1, 29.4)	28.3 (25.6, 31.4)^5^
QA, nmol/L	360 (344, 377)	349 (328, 373)	373 (348, 400)	357 (316, 403)	3	362 (345, 380)	355 (337, 373)
Other							
CRP, μg/L	2.26 (1.87, 2.72)	1.51 (1.17, 1.96)	1.26 (0.95, 1.68)^4^^,^^5^	2.29 (1.39, 3.77)	29	2.21 (1.83, 2.68)	2.28 (1.87, 2.79)
KTR, nmol/μmol	23.5 (23.0, 24.1)	23.0 (22.3, 23.8)	22.4 (21.7, 23.2)	22.7 (21.3, 24.1)	2	23.7 (23.1, 24.2)	23.2 (22.6, 23.8)
PAr	0.444 (0.413, 0.476)	0.500 (0.453, 0.552)	0.322 (0.289, 0.358)^3^^–^^6^	0.423 (0.351, 0.511)	18	0.429 (0.399, 0.461)	0.468 (0.434, 0.505)^5^
Creatinine, μmol/L	77.0 (74.6, 79.4)	72.4 (69.4, 75.6)	73.6 (70.2, 77.2)	71.4 (65.7, 77.5)	3	77.7 (75.2, 80.2)	76.0 (73.6, 78.6)^5^
HK:XA	3.24 (2.93, 3.58)	3.00 (2.61, 3.45)	2.69 (2.31, 3.14)	3.43 (2.63, 4.47)	10	3.36 (3.05, 3.71)	3.02 (2.73, 3.34)^5^
Cotinine, nmol/L	37 (30, 45)	48 (36, 63)	54 (41, 72)^3^	38 (23, 62)	19	38 (31, 47)	35 (28, 44)
Arginine, μmol/L	55.8 (43.2, 72.1)	97.3 (68.0, 139.0)	82.3 (55.2, 122.8)	72.4 (36.1, 145.2)	23	55.8 (43.0, 72.5)	57.1 (43.9, 74.2)
ADMA, μmol/L	0.529 (0.514, 0.544)	0.543 (0.521, 0.565)	0.576 (0.551, 0.602)^3^^–^^4^	0.546 (0.505, 0.590)	4	0.533 (0.517, 0.549)	0.525 (0.509, 0.542)
SDMA, μmol/L	0.571 (0.547, 0.596)	0.552 (0.520, 0.585)	0.629 (0.590, 0.672)^6^	0.604 (0.539, 0.676)	6	0.576 (0.552, 0.602)	0.569 (0.544, 0.594)
Homoarginine, μmol/L	1.82 (1.74, 1.91)	1.76 (1.65, 1.87)	2.28 (2.13, 2.44)^3^^–^^6^	2.05 (1.82, 2.31)	12	1.81 (1.72, 1.89)	1.86 (1.77, 1.95)
MMA, μmol/L	0.172 (0.162, 0.182)	0.170 (0.157, 0.184)	0.207 (0.189, 0.226)^3^^,^^5^^,^^6^	0.178 (0.152, 0.207)	9	0.176 (0.166, 0.187)	0.165 (0.155, 0.176)^5^

1 AA, anthranilic acid; ADMA, asymmetric dimethylarginine; BVS, B-vitamin score; CRP, C-reactive protein; FVS, fat-soluble vitamin score; HAA, 3-hydroxyanthranilic acid; HK, 3-hydroxykynurenine; KA, kynurenic acid; KTR, kynurenine:tryptophan ratio; MMA, methylmalonic acid; MV, multivitamin supplement; PA, 4-pyridoxic acid; PAr, 4-pyridoxic acid:(pyridoxal 5′-phosphate + pyridoxal); PLP, pyridoxal 5′-phosphate; QA, quinolinic acid; SDMA, symmetric dimethylarginine, tCys, total cysteine; tHcy, total homocysteine; tMet, total methionine; TVS, total vitamin score; XA, xanthurenic acid; αTOC, α-tocopherol; γTOC, γ-tocopherol.

2 Geometric means; 95% CIs in parentheses (all such values). Values by region were estimated with the use of mixed models that were adjusted for age, sex, and smoking status (former compared with never; current compared with never) with the cohort as a random effect.

3 Significantly different from the United States with MV use (*P* < 0.05).

4 Significantly different from the United States (*P* < 0.05)

5 Significantly different from United States with no MV use (*P* < 0.05).

6 Significantly different from the Nordic region (*P* < 0.05).

7 Sum of 25-hydroxyvitamin D_2_ and season-adjusted 25-hydroxyvitamin D_3_.

8 Significantly different from Australia (*P* < 0.05).

### Biomarkers and metabolites

The adjusted geometric mean (95% CI) biomarker concentrations for the geographic regions that were obtained from the mixed models are provided in Table 2 and for each cohort in Supplemental Tables 5 and 6. Across regions, the spread (CV) in adjusted geometric mean biomarker concentration was largest for folate (39%) and γ-tocopherol (38%), whereas the CV was <10% for 17 of 38 biomarkers. Folate was highest in the United States compared with in the other regions. Functional B-vitamin markers (tHcy, MMA, cystathionine, HK, and HK:XA) were generally high in the Asian region and low in the US region, and fat-soluble vitamins A, 25(OH)D, and α-tocopherol were higher in Nordic and Australian regions than in US and Asian regions.

Methionine and downstream OCM biomarkers were generally highest in the Asian region, whereas tryptophan and kynurenines were generally lowest in the United States and highest in Asia.

**FIGURE 3:**
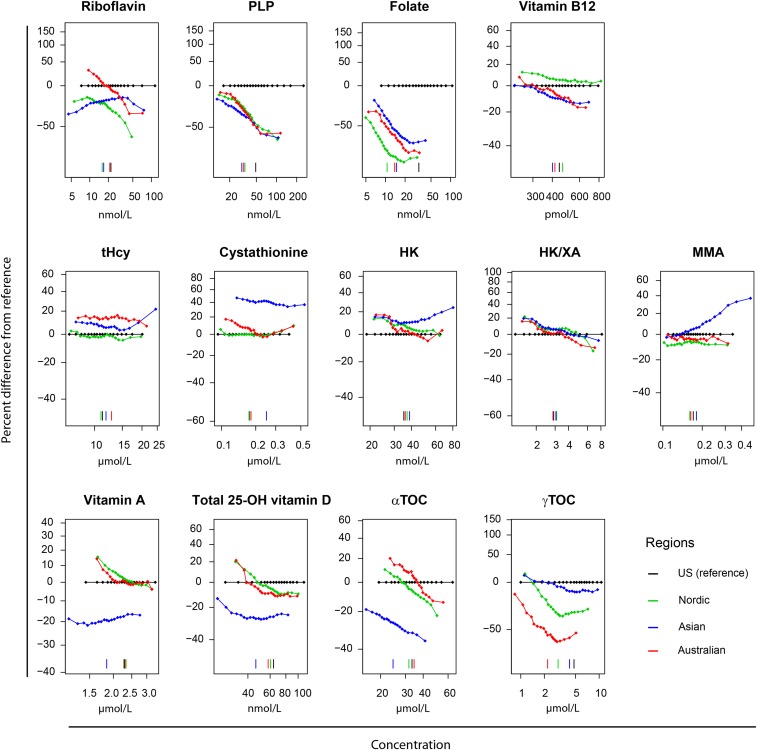
Distribution of vitamins and vitamin markers in regions from a quantile regression by 5th, 10th, 15th, 20th, 25th, 30th, 35th, 40th, 45th, 50th, 55th, 60th, 65th, 70th, 75th, 80th, 85th, 90th, and 95th percentiles. The models were adjusted for age, sex, smoking (former compared with never; current compared with never) and cohort. The *y* axis in each panel is scaled to show 3 SDs of the distribution of the biomarker in that panel. The vertical line in each panel indicates the 50th quantile for each group. HK, 3-hydroxykynurenine; HK/XA, 3-hydroxykynurenine:xanthurenic acid; MMA, methylmalonic acid; PLP, pyridoxal 5′-phosphate; Total 25(OH) vitamin D, 25-hydroxyvitamin D_2_ plus season-adjusted 25-hydroxyvitamin D_3_; tHcy, total homocysteine; US, United States; αTOC, α-tocopherol; γTOC, γ-tocopherol.

Quantile regression (**Figure 3**) showed that the entire distributions of PLP and folate and the upper part of the distribution of riboflavin were elevated in the United States compared with in the other regions. For tHcy, the entire distribution was lower in the US and Nordic regions than in the Asian and Australian regions, whereas the entire distribution of cystathionine was elevated in Asians. In Asia, the upper ranges of MMA and HK were higher than those observed in other regions. Compared with other regions, the distribution of the fat-soluble vitamins A, 25(OH)D, and α-tocopherol were lower in Asia, whereas γ-tocopherol was lower in Australia.

### Multivitamin-supplement use, mandatory folate fortification in the United States

For US multivitamin-supplement users and nonusers, the mean BVS was 0.80 (95% CI: 0.62, 0.97) and −0.01 (95% CI: −0.18, 0.17), respectively, the FVS was 0.41 (95% CI: 0.24, 0.57) and −0.13 (95% CI: −0.29, 0.03), respectively, and the TVS was 0.74 (95% CI: 0.59, 0.89) and −0.06 (95% CI: −0.21, 0.08), respectively (Table 2). US-based participants who were taking multivitamins had higher circulating concentrations throughout the concentration ranges of all vitamins (except for γ-tocopherol, which was lower), and had lower concentrations of all functional B-vitamin markers compared with US participants who did not use such supplements (**Supplemental Figure 3**).

US multivitamin-supplement users had a higher BVS than that in all other regions, a higher FVS than that of Asians, and a higher TVS than in Nordic and Asian regions (*P* < 0.05) (Table 2). PLP and folate were higher in US multivitamin-supplement users than in other regions (*P* < 0.05).

In US participants who were not using multivitamin supplements, the FVS was lower than in the Nordic region and higher than in Asia, folate was higher than in the Nordic and Asian regions, and γ-tocopherol was higher than in Asia and Australia (*P* < 0.05) (Table 2). The mean BVS tended to be slightly higher in US participants who were not using multivitamin supplements (−0.01; 95% CI: −0.18, 0.17) compared with Nordic and Asian regions [−0.28 (95% CI: −0.53, −0.03) and −0.25 (95% CI: −0.53, 0.02), respectively], although these differences were NS.

In the United States, the circulating geometric mean concentration of folate before and after the implementation of the mandatory folate fortification of flour (1998) was 23.9 nmol/L (95% CI: 19.1, 29.8 nmol/L) compared with 37.2 nmol/L (95% CI: 29.6, 46.7 nmol/L) (*P* < 0.001). No other biomarkers were different before compared with after folate fortification (data not shown). For tHcy, the geometric mean was 12.1 μmol/L (95% CI: 11.1, 13.2 μmol/L) compared with 11.5 μmol/L (95% CI: 10.5, 12.6 μmol/L) (*P* = 0.84).

### Vitamin deficiency

Plasma and serum concentration cutoffs that were used to indicate vitamin deficiency were 5 nmol/L for riboflavin (14, 32), 20 nmol/L for PLP (29), 5 nmol/L for folate (33), 150 pmol/L for vitamin B-12 (33), 0.7 μmol/L for vitamin A (34), 30 nmol/L for 25(OH)D (35), and 12 μmol/L for α-tocopherol (36).

Vitamin B-6 deficiency was observed in 16.1% of the total population (**Supplemental Table 7**) and ranged from 9.4% in US participants to 23.5% in Asians. Of the total population, 9.1% were vitamin D deficient with the highest prevalence of 14.7% in the Asian region and the lowest prevalence in Australian (4.5%) and Nordic (4.8%) regions. For the other vitamins, the prevalence of deficiency was low. In US multivitamin-supplement users, the highest prevalence of deficiency was 3.3%, which was observed for vitamin B-6 and vitamin D, compared with 12.7% and 9.5%, respectively, in US participants who were not taking multivitamin supplements.

## DISCUSSION

### Principal findings

We report circulating concentrations of 38 biomarkers that are related to vitamin status, one-carbon metabolism, and tryptophan metabolism in 5314 healthy individuals from 20 cohorts who represented US, Nordic, Asian, and Australian regions. Biochemical analyses were performed by a single laboratory, which enabled the direct comparison of biomarker concentrations across regions and cohorts. Composite vitamin scores that were based on serum and plasma vitamin concentrations showed that the general B-vitamin status was highest in the United States, and the general vitamin concentration was low in the Asian region. We observed a high general vitamin status in multivitamin-supplement users in the United States. Differences in B-vitamin concentrations were further reflected by the concentrations of one-carbon and tryptophan metabolites, which serve as functional markers of B-vitamin status.

### Vitamin status across regions

The grouping of the 7 measured vitamins, which reflected general positive intercorrelations, combined with the similar location of the US cohorts in the space spanned by the first 2 PCs from the PCA motivated the construction of circulating vitamin scores.

The grouping of US cohorts away from Nordic, Asian, and Australian cohorts in the score plot combined with the grouping of the vitamins in the loading plot probably reflected abundant fortification, enrichment, and dietary supplementation practices in the United States. For instance, the BVS was higher in the United States than in the other regions. The consumption of fortified foods strongly affects B-vitamin status in the United States (5, 8) with ready-to-eat cereals, which are a staple food in the United States, being a major source of many vitamins (37–40). Furthermore, widespread voluntary fortification practices in the United States are not bound by strict regulations and legislation, which often result in the fortification of food products above mandatory concentrations (5).

At the time of blood sampling, vitamin fortification was much less common in the other regions included in this study, which is in accordance with the lower vitamin concentrations that were observed for these regions. Within the Nordic region, Norway (41) and Sweden (42), fortification was limited to only vitamins A and D in dairy products, and the consumption of cod-liver oil provided a further source of vitamin D in Norway (43), whereas there was no fortification in Finland (44). The concentrations of vitamins B-2, B-6, folate, and B-12 in the Nordic cohorts that were included in LC3 were similar to the concentrations that were previously measured by the same laboratory in Northern European (Sweden and Denmark) (45) and Norwegian (14, 46) cohorts. In Asia, China had no vitamin fortification during the sampling period, whereas Singapore (47) started fortification with folic acid in 1998, and in Australia, only the fortification of cereals with retinol took place (48). In addition, it has been reported that intake of vitamin A is low in China (49) compared with in the United States (9, 50). Low circulating 25(OH)D in Asians (51, 52) was suggested to be related to skin pigmentation and the cultural avoidance of sun exposure (52).

### Multivitamin-supplement use

The high prevalence of multivitamin-supplement use among US participants in this study (36.8%) (Table 1) is consistent with the literature (53, 54) and contributed significantly to the high B-vitamin status in this region. For populations outside of the United States in this study, multivitamin-supplement use was less common, and data were available for only a few of these participants. A prevalence of multivitamin-supplement use of 22–27% in the Nordic countries Sweden and Norway has been reported (55), similar to the 26.8% shown in the current study. Because of the limited data available, stratification by multivitamin-supplement use in Nordic, Asian, and Australian regions was not performed when modeling vitamin status. Thus, the vitamin status obtained for each of these regions was influenced by the inclusion of participants who were not using supplements as well as subjects who were using supplements. Despite the reported increases in use of vitamin supplements over the past decades (56, 57), we found that the year of blood sample collection was not related to vitamin status (data not shown). This result might be related to the considerable heterogeneity of the participating cohorts or the distribution of participants over the time span during which blood samples were collected.

Notably, US multivitamin-supplement users showed distributions in the upper concentration range for all vitamins except for γ-tocopherol, which was lower. A study of middle-aged Americans showed that supplement users, on average, had vitamin intakes that were considerably higher than the estimated average requirements for several vitamins (58). It has also been reported that, in some US subgroups, supplement use has provided vitamins that are already consumed in adequate amounts (9, 59).

### B-vitamin status and one-carbon and tryptophan metabolites

Components of OCM and tryptophan metabolism may serve as functional markers of B-vitamin status with inverse relations observed for tHcy with folate and vitamin B-12 (60); for cystathionine, serine, HK, and HK:XA with vitamin B-6 (29); and for MMA with vitamin B-12 (60). The grouping of OCM biomarkers HK and HK:XA opposite the B vitamins in the loading plot was in-line with these established relations and likely reflected the functions of B vitamins as cofactors of enzymes that are involved in these metabolic pathways (61). The low concentrations of functional markers in the US cohorts were in accordance with high circulating concentrations of individual B vitamins. A notable exception was observed for tHcy, and a lack of strong inverse relations between geometric means of tHcy with folate across geographic regions in Europe has been reported (45). In contrast, in studies in homogenous populations, intakes of folate (12, 62), vitamin B-2 (12), and vitamin B-6 (12) and circulating concentrations of folate (15, 62), vitamin B-12 (15, 62), vitamin B-2 (15), and vitamin B-6 (15, 62) were inversely related to serum and plasma tHcy concentrations. A lack of inverse associations between tHcy and B-vitamin status across geographical regions may be related to ethnicity, genetics, nutritional, and other lifestyle factors that are known to influence tHcy (63).

Circulating concentrations of the tryptophan metabolite HK (3) and the HK:XA ratio (64) have been shown to be functional markers of vitamin B-6 status, and both markers were inversely related to PLP across cohorts in the current study. The HK:XA ratio has been suggested to be a better functional vitamin B-6 marker than HK alone (64), but the HK:XA was not the lowest in the United States, which was the population that showed the highest PLP concentrations. Kynurenines other than HK were also related, although more weakly than HK was, to circulating concentrations of riboflavin and PLP in a homogenous Norwegian population (65) and also across cohorts in this study. However, we did not consistently find the anticipated relations across regions. Conceivably, as with tHcy, additional factors may influence circulating concentrations of tryptophan metabolites.

### Strengths and weaknesses

The study has a large sample size, particularly from the United States. Information on multivitamin-supplement use in the US cohorts enabled the categorization according to this influential behavior.

A further strength of this work is the use of a single laboratory for the analyses of all biomarkers and metabolites. Between-batch CVs of quality-control samples showed that the variability of the assays that were used was small (**Supplemental Table 8**). Inadequate sample handling or storage will increase serum and plasma concentrations of MetSO, choline, and anthranilic acid combined with decreased methionine, HK, and 3-hydroxyanthranilic acid (26, 66) and may compromise the usefulness of comparing specimens from different biobanks. We did not observe biomarker concentrations that indicated detrimental sample handling or storage in this study.

Limitations of the study also exist. Because the consortium was formed to investigate lung cancer, it was oversampled with regard to the number of smokers. However, all models were adjusted for smoking. The included Asian cohorts consisted of Chinese subjects only. Thus, the current data cannot be expected to be representative of the Asian region. The Australian cohort was rather small. Information on multivitamin-supplement use was available for relatively few participants from the Nordic and Asian regions. Information on the use of single vitamin supplements was available for very few participants and, thus, was not included in this work. Most participants were middle aged or older, and the reported data may not be representative of other age groups. Finally, information on fasting status, which influences the circulating concentrations of some biomarkers (67), was not available for all cohorts and, therefore, was not included in the analyses.

In conclusion, the present study is the first study, to our knowledge, to robustly and comprehensively investigate vitamin status as reflected by circulating biomarkers in different regions from around the globe. Through the quantification of circulating concentrations of 7 vitamins and 5 functional B-vitamin markers, we show that B-vitamin status was higher in the United States than in the Nordic region, Asia, and Australia, whereas vitamin status, in general, was low in the Asians. The high prevalence of multivitamin-supplement use is suggested to be a major determinant of the higher B-vitamin status in the United States. The presented data will be useful when investigating circulating biomarker concentrations, nutritional status, and risk of chronic diseases particularly when different regions or populations are compared.

## Footnotes

39 Abbreviations used: BVS, B-vitamin score; FVS, fat-soluble vitamin score; HK, 3-hydroxykynurenine; LC3, Lung Cancer Cohort Consortium; MetSO, methionine sulfoxide; MMA, methylmalonic acid; OCM, one-carbon metabolism; PCA, principal component analysis; PLP, pyridoxal 5′-phosphate; tHcy, total homocysteine; TVS, total vitamin score; XA, xanthurenic acid; 25(OH)D, 25-hydroxyvitamin D.
